# Intersecting challenges and ways forward: The impact of the COVID-19 pandemic on an urban First Nations community in Southern Ontario, Canada

**DOI:** 10.1371/journal.pone.0335020

**Published:** 2025-10-27

**Authors:** Eric N. Liberda, Fatima Ahmed, Nicholas D. Spence, Sarah Plain, Robert J. Moriarity, Leonard J. S. Tsuji, Nadia A. Charania

**Affiliations:** 1 School of Occupational and Public Health, Toronto Metropolitan University, Toronto, Ontario, Canada; 2 Department of Physical and Environmental Sciences, University of Toronto, Toronto, Ontario, Canada; 3 Aamjiwnaang First Nation, Sarnia, Ontario, Canada; 4 Department of Public Health, Auckland University of Technology, Auckland, New Zealand; University of Saskatchewan, CANADA

## Abstract

The COVID-19 pandemic has had wide-ranging impacts on communities worldwide, with Indigenous communities in southern Ontario, Canada, being no exception. Partnering with Aamjiwnaang First Nation, we explored the multifaceted impacts of the pandemic and learnings for the future. This study utilized semi-structured interviews with the community’s pandemic committee and other front line essential services (N = 12) to explore the nuanced dimensions of the pandemic’s effects. Data were analysed using a template approach to codebook thematic analysis to examine various aspects of the pandemic response. Five main themes were identified, including: (i) Wellbeing and mental health, (ii) Work-life balance, (iii) Community and social factors, (iv) Organizational dynamics, and (v) Lessons learned and future planning. Our findings unveiled a multifaceted spectrum of challenges, encompassing socioeconomic, psychological, and organizational aspects, which the First Nations community encountered amidst the pandemic. Despite these challenges, the commitment to community adaptation and collaboration highlighted the resilience cultivated through strong Indigenous leadership, trusting partnerships, and transparent communication, contributing to an effective response. This research stresses the need for future pandemic preparedness efforts to prioritize Indigenous leadership and address the social and cultural determinants of Indigenous health. Additionally, to effectively address future environmental and health emergencies, there is a pressing need to adopt an all-hazards approach and develop comprehensive, yet adaptable plans tailored to meet the diverse needs of communities.

## Introduction

The global outbreak of COVID-19 has served as a landmark event in modern history, profoundly impacting every facet of human society and requiring quick and adaptive responses across all sectors. Recent data indicates the toll of the pandemic, with over 774 million infections and 7 million deaths worldwide [1]. Yet, the pandemic’s impact has not been uniformly distributed, disproportionately affecting Indigenous communities [2–4] and exacerbating existing social inequities [2]. For Indigenous Peoples, the historical and ongoing colonial context has embedded systems of social inequity, profoundly affecting the vulnerability of many communities to major public emergencies such as the COVID-19 pandemic [2,3]

Indigenous communities across the globe faced unique challenges during the COVID-19 pandemic. In many cases, they experienced higher infection rates and more severe outcomes due to pre-existing health disparities, limited access to healthcare services, overcrowded living conditions, and inadequate infrastructure [5]. Additionally, cultural practices such as communal gatherings and ceremonies, which are integral to Indigenous identity and well-being, were disrupted by public health measures such as lockdowns and social distancing, further exacerbating social isolation and mental health concerns [6].

Reserves, herein referred to as “Indigenous or First Nations communities,” provide a critical context to examine the lived experiences associated with the pandemic. Reserves encompass a diverse range of social, geographical, historical, and cultural attributes across Canada [7]. Moreover, numerous programs and policies formulated on reserves directly impact residents’ day-to-day lives, including pandemic emergency planning [7]. Therefore, identifying and addressing factors to mitigate the impact of pandemics in the future is essential to maximize the health and well-being of Indigenous Peoples and reduce social inequities [2,3]. Structural inequities, including limited access to healthcare, isolation, remoteness (in some cases), and food insecurity, further compound the challenges faced by many Indigenous communities, underscoring the urgency for studies that focus on these specific impacts and offer perspectives critical for a holistic understanding of the pandemic’s effects. Being prepared for a pandemic is vital to mitigate ensuing risks, particularly in First Nation communities grappling with existing vulnerabilities [3].

The context of Indigenous communities in relation to public health emergencies is multifaceted and deeply rooted in historical, social, and cultural factors. It is widely recognized that Indigenous communities in Canada bear the enduring legacy of colonization and forced assimilation, which have left indelible impacts on their health and well-being [8,9]. These historical factors have caused a lack of trust in mainstream healthcare services, creating systemic barriers to healthcare access, and resulting in poorer health outcomes [10]. Culturally, communities possess their own Indigenous knowledge systems and healthcare practices, often overlooked, or misunderstood by mainstream healthcare providers [11]. This cultural gap can lead to ineffective or culturally inappropriate healthcare interventions [12], hindering effective communication. Therefore, pandemic plans must be informed by Indigenous peoples, recognizing, and respecting their diverse needs and cultural practices. Such plans should incorporate culturally- and community-appropriate public health and control measures, addressing underlying social and cultural determinants [13–16]. However, there remains a need for the generation, evaluation, and updating of local pandemic plans with Indigenous communities to ensure they adequately address the local context and are culturally appropriate, emphasizing the role of structurally marginalized groups in creating pandemic plans that properly address and protect their rights [3,14,17–22].

Experiences during the H1N1 influenza pandemic in 2009 exposed significant gaps in pandemic preparedness and response for Indigenous communities [17,19,20]. Globally, the COVID-19 pandemic response by governments has been criticized for being reactive and inadequate to address the needs of Indigenous communities [3]. Consequently, many Indigenous communities developed and implemented their own protective measures, highlighting the importance of Indigenous autonomy and self-determination [3,14,18]. Previous research has underscored the importance of Indigenous engagement and leadership in developing appropriately tailored preparedness and response efforts [13–16,23]. Collectively, this underscores the necessity of adopting a multi-dimensional and culturally appropriate approach to public health emergency planning and mitigation measures for Indigenous communities.

The intricate nuances affecting Indigenous communities during the COVID-19 pandemic transcends statistics and epidemiological data- exploring the realm of lived experiences, cultural norms, and deeply rooted historical contexts. This complexity necessitates a nuanced examination of Indigenous communities’ experiences during the pandemic to identify gaps in pandemic preparedness and response and understand the unique successes achieved. Therefore, examining the experiences of Indigenous communities, particularly the pandemic committee, is essential. It provides valuable insights into the effectiveness of existing pandemic plans and measures, including culturally specific interventions and community-driven initiatives, essential for refining public health strategies. Moreover, a thorough examination can highlight the resilience and innovative coping mechanisms employed by Indigenous communities, offering learnable strategies for integration into broader public health initiatives and the transfer of Indigenous knowledge into action [24,25]. Understanding the successes and challenges faced by Indigenous communities during the COVID-19 pandemic offers a comprehensive evaluation of public health measures, from risk communication to vaccine distribution. These insights have real-world implications for enhancing the efficacy and cultural appropriateness of healthcare interventions, not only for future pandemics but also for ongoing public health challenges [26].

While the impacts of intergenerational trauma, residential schools, and broad mental-health and social effects of COVID-19 on First Nations people have been described [27], there is less real-time operational evidence from urban First Nations about how Indigenous-led committees and frontline teams implemented decision-making, communications, and vaccine programs [3,28]. This study provides that perspective for an urban community in southern Ontario, describing concrete practices (trusted, non-coercive messaging through community channels; partnership governance with the local public health unit; rapid food and technology supports) and jurisdictional frictions that affected implementation. By examining the pandemic committee and essential services’ experience shortly after the acute phase (March 2023), we provide post-event, practice-level lessons that complement prior remote-community and population-level accounts. We also translate these lessons to the WHO Health EDRM/PRET ‘all-hazards’ planning frames to make them portable for future respiratory threats [29].

## Methods

The study took place in Aamjiwnaang First Nation (AFN), a community comprising of approximately 2500 registered Chippewa First Nations Peoples (approximately 900 residents living on-reserve) [30], with Ojibwa as their heritage language [31]. The name “Aamjiwnaang,” meaning “at the spawning stream,” reflects the community’s location on the St. Clair River within Sarnia’s city limits in southern Ontario, Canada [31]. AFN operates a kindergarten to Grade 2 on-reserve school; from Grade 3, children attend Lambton Kent District School Board schools. Pandemic response was led by a central working group (elected leadership; Health Director/Health Centre; emergency management; education; public works; administration) that collaborated with outside partners on vaccine logistics and public-health guidance. This is consistent with a WHO ‘whole-of-society’ approach to emergency coordination as well as Indigenous-led implementation models [29].

Prior to the COVID-19 pandemic, AFN had ongoing connections with Lambton Public Health (LPH; e.g., communicable-disease and school-health liaison), the regional hospital system for acute-care needs, and had protocols in place with the Lambton Kent District School Board. The established relationships were leveraged to co-plan and co-staff the on-reserve COVID-19 vaccination clinics and the screening/communications alignment, and enabled a smoother rollout than might otherwise have been the case. Pandemic operations therefore involved tri-partite coordination (AFN, LPH, and Indigenous Services Canada (ISC)) to integrate community-run, culturally safe communications and clinic workflows with provincial eligibility rules and federal supports; AFN publicly announced a joint rollout with LPH and ISC in February 2021 [32,33]. These jurisdictional arrangements and community-led services stand in contrast to neighbouring small cities, which are served exclusively by provincial/municipal systems [34].

Our research employed a community-based approach, aligning with the study’s objective of understanding the community’s pandemic experience. Such approaches, common in pandemic planning help develop tailored pandemic plans and responses in partnership with Indigenous populations [13–16,18,23,35].The presented study builds upon a long-standing community-academic partnership in which community members and researchers have collaboratively addressed locally relevant environment and health issues, now extending our focus to the COVID-19 pandemic. Our aim was to understand the experiences of implementing mitigation measures and explore strategies for improvement to guide future pandemic planning efforts. This information will ultimately be used to update local pandemic plans following a collaborative cyclic planning process.

### Study participants and data collection

Under the guidance of the community Health Director, we identified and recruited members of the pandemic committee and essential workers pivotal during the pandemic to take part in semi-structured interviews between March 1^^st^^ and March 20^^th^^, 2023. Collaborating with community leaders and our academic research team, we developed an interview guide exploring various aspects of the COVID-19 experience, including community infection control measures, surveillance, healthcare services, and communication strategies. Prior to participation, written informed consent was obtained from all participants, ensuring confidentiality throughout the process.

In total, 12 in-person interviews were conducted, each lasting approximately 30 minutes to one hour. All interviews were audio-recorded and transcribed verbatim for subsequent analysis. Ethics approval for the research was obtained from the research ethics boards of Toronto Metropolitan University (REB-2022–221), the University of Toronto (Protocol #00044366), and Auckland University of Technology (#23/76).

### Data analysis

We employed a template approach to codebook thematic analysis, which enables a structured yet iterative approach to the coding of textual data and conceptualisation of themes [36]. The template codebook approach incorporates various levels of coding granularity thereby enabling the capture of rich and detailed aspects of the data [36]. Transcripts were initially read and then re-read to familiarize ourselves with the data. Initial coding involved labelling chunks of raw data to codes and was predominantly inductive (data-driven) to focus on the experiences and perspectives of the participants. At the request of the community, participants were coded with letters instead of numbers. The codebook was developed based on a subset of data that defined each code and included participant quotes as examples. The codebook was subsequently applied to the entire dataset, refining it as needed. The codes were then grouped into overarching themes which offer a multi-faceted understanding of participants’ pandemic planning and response.

## Results

### Well-being and mental health

This theme explores the profound and lasting effects of the COVID-19 pandemic on well-being and mental health, as discussed by participants.

Many participants highlighted the pandemic’s impact on food security, as people were unable to work. According to one participant, a pre-pandemic community survey revealed a food insecurity rate of nearly 50%, a figure they say was likely worsened during the pandemic. This concern was echoed by another participant,

*“I think every First Nation was doing that [food programs] because we had to make sure that people were able to get their groceries… a lot of people were worried about food security… So, I think just making sure that people are aware that they’re not going to be abandoned and we’re not going to forget about them… I think that’s most important.”* (Participant L)

During times of isolation, community members received porch drop-offs of vital supplies including food, water, and medication. Furthermore, local leadership distributed gift cards to assist with grocery needs, extending support to vulnerable community members. In a tailored adaptation of the Meals on Wheels initiative, staff delivered meals to those in need while also conducting regular check-ins. To nurture community bonds, families were supplied with meal kits, and an online cooking program facilitated collective cooking experiences. These supports were centralized initiatives that were organized by the AFN Health Centre as opposed to an ad-hoc, word-of-mouth method. For instance, teams pre-arranged porch drop-offs of food/water/medications, delivered grocery gift cards, and pivoted the Meals on Wheels program to have staff bring hot meals to Elders in conjunction with wellness checks (i.e., safety calls/doorstep check-ins). These needs were magnified in a First Nations context through higher baseline food insecurity and persistent connectivity gaps that hindered virtual care and social support. Additional initiatives were also implemented to alleviate food security concerns,

*“Food security needs increased a lot... So, we’ve put a lot of that [COVID funding] towards the food security initiatives… We have a food bank… but the numbers increased like a lot… We started doing a produce giveaway where we buy produce boxes from a produce company in town, and then we still do it the same [drive through pickup] … We also started doing an additional grocery giveaway… The produce boxes were hitting like anybody in the community that wants that, but the grocery giveaway, it’s really only the people that really need the extra help…So once a month they just they do the fresh stuff… We’re getting about 45 households coming through and everyone’s really good with like only taking a few things and making sure that they’re leaving enough food because they can see all the families waiting in line behind them.”* (Participant H)

This participant also shared a past attempt at establishing a community garden, which failed due to insufficient capacity for maintenance. However, community members had an interest in cultivating their own home gardens, “*That was actually something that really picked up. The band bought topsoil and had public works going out and rototilling for people… There was a lot of uptake during that first year.*” (Participant H). These perspectives underscore food security as a notable challenge during the pandemic, sparking the rise of community-driven initiatives crucial for addressing immediate needs.

During the COVID-19 pandemic, participants described the considerable toll on **mental health**, highlighting the emotional and psychological challenges they encountered. Lockdown measures, which resulted in isolation, proved particularly detrimental given the significance of personal and environmental connections within First Nations communities. Participants observed the ripple effects of these lockdown measures:

“*I don’t think were made to be isolated… We’re social beings and I think it they had a huge impact on our mental health and addictions*” (Participant D);

*“Some of our substance use clients… who had solid foundation for recovery…they had relapsed… That was a pretty sad impact for our team… Especially because those people were doing really well.”* (Participant L)

Participants expressed the strain of managing heavy caseloads while providing essential emotional support, especially crucial for youth facing heightened anxiety and depression amid lockdowns and limited resources:

“*That preteen into their teenage years… Their ability to like self regulate social situations. Their anxiety… mental health as we know, it’s just really bad”* (Participant K);

*“Some of the students do have long term health impacts because of that [lockdowns] and they may have left school early because of it, not completed their education… Our staff does a really good job of checking in… They’re all, you know, communicating with each other and trying to wrap supports around students… So that they do understand that there are supports out there for them to get the help that they need to change their health outcomes around in that not let it be a barrier to their success.”* (Participant I)

During the pandemic, there was a significant increase in the use of Jordan’s Principle, a program providing funding for diverse health, social, and educational needs. Participants noted a rise in applications for both Jordan’s Principle and for addressing complex needs and traumatic events. Moreover, in response to mental health challenges, a shift to online platforms occurred, introducing various initiatives such as men’s mental health support groups, women’s wellness groups, online sessions with traditional healers, virtual counseling services, wellness checks for students, and additional self-care resources via the health center’s online platform. Continued mental health support was more effective in-person for engagement of care and complex needs (where possible), while virtual visits were helpful in maintaining engagement in stable patients with reliable internet access. While these online supports were beneficial for many community members, they also presented logistical and technological challenges for some:

*“I know that there were people who struggled with isolation, particularly mental health clients who are used to… kind of going and visiting their supports and in person”* (Participant L);

*“If they didn’t have access to like technology… it was hard for families… We often see kiddos with like without their parents… When we started seeing them virtually like you really do need a parent in the room. So, like just their mental capacity in order to participate with their parents, or with their kiddo… it was difficult for families”* (Participant K);

*“I think it really impacted our students who were made to work from their laptops or their computers at home. Some of them didn’t have the technology. Some of them didn’t have the Internet, so there were those impacts that were impeding on their ability to contribute or participate in education… Mostly the school board contributed the laptops or Chromebooks that were needed for the school. We used iPads in our school…We had to figure it out like it was a learning process.”* (Participant I)

While the public health orders were similar across the country, the effects of the lockdown measures were not equal for First Nations. Pre-existing inequities, including on-reserve food insecurity, crowded or unsuitable housing, and lack of broadband, further compounded social isolation, disrupted schooling and virtual care, and increased risk of relapse or overdose. There were also frictions in coordination in urban jurisdictions. At the same time, many Indigenous nations leveraged self-determination (e.g., checkpoints) to mitigate against early transmission. These narratives underscore the critical need for continual mental health support, emphasizing proactive measures to confront evolving community challenges.

### Work-life balance

Participants described the pandemic’s profound impact on work-life balance, particularly the difficulties of switching between remote work and returning to the office post-restrictions. Managing work, family, and community duties proved challenging, accentuated by the repercussions of mitigation efforts. These participants also emphazied that remote work took a toll on mental health, fostering feelings of isolation. These experiences highlight the necessity for adaptable and supportive measures amid unprecedented circumstances. Staff also shared instances of burnout, emphasizing the heightened importance of mental health support,

*“My main thing was to be there to be a support for them [staff]… I would say [mental health] increased from the beginning to the end. I feel like that’s way more of a high priority than it was before … I’m trying to get staff involved more… Do events and things… get everyone back together as a community because I feel like we lost that for a little bit.”* (Participant G)

Though work–life pressures were common during the COVID-19 pandemic, the extent of impacts in this First Nations community was different to those in nearby non-Indigenous populations. Indigenous workers were more likely to have less-flexible on-site jobs and to experience childcare/school disruptions, crowded housing, and connectivity gaps that limited ability to telework and home-school children and to heighten burnout. These contextual factors may help explain the greater degree of strain described by participants. Collectively, these comments highlight the complex, interrelated impacts of the pandemic among families in this First Nation community and raise concerns about the long-term impact on their health and well-being.

### Community and social factors

This theme explores into the pandemic’s impact on the community and their response, emphasizing mutual care, local leadership, and the importance of social ties’ while addressing systemic issues.

Many participants in the community discussed a shared goal of caring for each other and safeguarding the most vulnerable,

*“Being a First Nation community [means] that we’re vulnerable in all sectors for health… We have a lot of people within the community that were… high risk for vulnerability, for illnesses. I think people were respectful for getting [and] the wearing [of] PPE, stay[ing] in their social distancing and you know taking the vaccines.”* (Participant J)

Participants stressed transparent communication as crucial, emphasizing the importance of reaching out to isolated individuals and reassuring them of available support. Trust is important, but acceptance of messages also depends on clear communication about uncertainty, messengers that are deemed credible, timing, consistency, cultural safety, and steps to action. Clear communication about uncertainty, even when it is quite high, does not necessarily damage trust and may in fact maintain it. The directives for managing the pandemic, guided by community leaders, covered various aspects from communication strategies to the vaccine program. This approach aimed to foster trust and effectively meet the community’s specific needs:

*“They [community members] trust us and they look to us to get them that vaccine… Whereas if they had to go into Sarnia, it would be totally different…They’re friendly at public health, but it’s just different and it’s within your own community”* (Participant B);

*“I think in terms of what we did for promotion… Everybody did like just taking pictures of people getting it [vaccine], you know, a familiar face, a trusted face, hopefully and just kind of, you know, sharing it and promoting it that way.”* (Participant F)

These factors were vital as vaccine mis/dis-information and conspiracy theories were prevalent. For instance, one participant shared that some community members wanted to get vaccinated off-reserve due to previous traumas and systemic issues within healthcare, particularly concerning Indigenous populations,

*“There were certain individuals that…feel like in the past the government has tried to wipe out the nation… the theory of maybe they’re sending us bad vaccines going down [to Sarnia] instead.”* (Participant C)

For this reason, participant narratives emphasized agency, particularly in vaccine decision-making. The community’s approach was transparent and non-coercive, offering choices and prioritizing at-risk individuals. At the same time, a number of provincial and federal messages were sending signals about enforcement or conditional access that some participants contrasted with the local non-coercive approach (e.g., Ontario’s proof-of-vaccination policy for certain indoor settings, and enhanced enforcement of the stay-at-home order; federally, mandatory vaccination for federal public servants and for air/rail travellers). On-reserve health staff adapted guidelines to prioritize vaccinating those most at-risk, provided additional information to support vaccine decision-making for children, and offered options if community members wanted to be vaccinated elsewhere. This stood in contrast to the government’s contrasting approach, as expressed by one participant,

*“Educate people about it [the vaccine] and not be forceful, because I felt like the government was really forcing everything. And it shouldn’t have been that way. And they might say we’re just trying to control the spread, but I don’t know.”* (Participant B)

Participants highlighted the potential benefits of offering vaccination services directly within the community, especially on the reserve, to alleviate concerns and enhance comfort levels among residents. This emphasizes the need for targeted communication and education efforts to address vaccine hesitancy and rebuild trust within communities.

During the discussions, efforts to support the community’s needs were highlighted. One of these needs were sustaining vital social connections which were integral to the community’s overall well-being,

*“First Nation people we love to be together. We love to gather. We love our families, and we need to be with them to be healthy … The pandemic was very, very difficult for us… I feel that it really impacted our community, and it impacted a lot of… our youth more so…because of not being able to have that interaction…we need in order to be well balanced and happy and healthy.”* (Participant A)

To address this, innovative solutions such as outdoor programs and virtual engagements like contests, bingo, book clubs, and art kit giveaways were implemented to foster connectivity and overall well-being. The shift to online platforms enabled the community to include individuals who might not have had access otherwise, as noted by one participant, *“There’s people all the way who knows where, could live hours away that would never have an opportunity to participate in any of our programs, but they were joining things online.”* (Participant H)

Moreover, to ensure community members were informed about available supports, information packages were disseminated. These packages contained contact details for resources, including mental health workers. Additionally, frequent updates were shared on the community’s social media platforms and in the community newsletter, known as *The Chippewa Tribe-Une.*

Despite these efforts, some community members encountered challenges in accessing supports. At times, staff also faced difficulties in adequately meeting the needs of community members:

*“That was happening because they didn’t have phones…they didn’t have Internet, so they probably felt like they were falling through the cracks”* (Participant E);

*“I prefer in-person working face-to-face with individuals. Because it just makes it a lot easier for doing your observations, seeing what their needs are, seeing what their living conditions are, especially if it was somebody brand new that came on to services. You don’t know what their home environment was really like.”* (Participant C)

During the pandemic, another social challenge in the community arose from restrictions on funeral services, making it difficult to ensure community members felt adequately supported during times of grief. As discussed by some participants:

*“You feel you put your health in jeopardy going to the funeral, but you want to be there, so that’s a lot of mental health there”* (Participant B);

*“We had no funeral [s]…. We could only invite like 25 or so… So, you had no visitation, but it really impacted it because it you didn’t have that connection that you that you needed”* (Participant A);

*“In the very beginning, we were strictly, we weren’t even hosting funerals here, and we were strictly doing whatever the Funeral Home was doing and then eventually started to do things a little differently here …Even that [in person funerals], I feel like we relaxed a little bit more than what the rules were off reserve once we started doing in person, but then we kept things like masking and hand sanitizer in place and just put out extra notices to the community.”* (Participant H)

The pandemic posed challenges to the community’s social cohesion, notably seen in the constraints on funeral services. Despite these limitations, the community innovated by organizing outdoor services while ensuring safety measures. Additionally, community members expressed a desire to apply similar adaptive strategies to address exacerbated systemic issues, including unemployment among vulnerable members unable to work during the pandemic,

*“You can probably see it in within the city of Sarnia even now, like the homelessness… A lot of people lost their job, lost their house… I think that was a huge problem we are still dealing with today…Everybody’s trying to figure out in Aamjiwnaang here, like how we can build more houses and build more affordable housing …even our seniors looking for places to live… It’s still the effects of COVID, I believe, and the way the economy is, you know, it’s just everything so expensive here now.*” (Participant J)

Moving forward, these experiences will continue to inform and strengthen the community’s response to future challenges, fostering a deeper sense of unity and resilience among its members.

### Organizational dynamics

The theme of organizational dynamics explores how workplaces adapted during the pandemic, with proximity to Sarnia providing valuable resources and integration into broader response efforts. One participant highlighted adaptability, efficiently utilizing existing infrastructure to overcome challenges,

“*We already had laptops we were already using, and getting ready to use the EMR (emergency medical records) … so I felt like we were able to transition pretty seamlessly. We had to change PPE (personal protective equipment) practices and all this stuff all the time, but I feel like we really went with the flow and we were able to do everything.*” (Participant G)

Collaboration with the local public health unit was vital in disseminating information and allocating resources, particularly seen in the successful vaccine rollout,

“*Our vaccine rollout was in partnership with Lambton Public Health … they were great to work with. Seamless for the most part, our rollout went pretty smoothly in terms of…the setup and the promotion and you know getting people signed up… partnering with them was a huge help”* (Participant F).

This partnership highlighted effective coordination and mutual support, echoing sentiments of collaboration among participants. Concurrently, leveraging existing communication channels like email and social media played a crucial role in reaching diverse demographics and promoting adherence to mitigation measures, as highlighted by one participant,

*“Communications was the key…I can’t stress enough how well we worked because we had the communications that we had… our phone system already connected to our e-mail. So, we could get information right away… We were still able to help the community when we needed to.”* (Participant E)

Despite the resilience exhibited by staff and community members, persistent challenges emerged, notably concerning fairness in workload distribution and financial compensation. While participants generally conveyed a positive tone regarding community adaptation and teamwork, they also addressed the obstacles they encountered. Several participants emphasized the importance of enhancing the community’s business continuity plan to ensure equitable distribution of capacity across essential organizations and departments, such as the food bank and mental health services. This would help ensure essential services and other staff are adequately supported. Moreover, a participant spoke to differences in renumeration depending on their jurisdiction,

*“Provincially, the nurses …were supposed to get a $5000 from the Ford government… but only the Provincial nurses got and the Federal nurses did not, and nurses on Reserve are Federal. That made me feel like, what did I do then… they [federal nurse consultants] never even got acknowledged. That’s discrimination.”* (Participant B)

Similarly, other participants also spoke of the lack of financial compensation stating,

*“I think it is very important in order to recognize them [staff] and that’s… financially… because so often people like them go unnoticed. And I think that if any time we should have seen how heavily you have to rely on them and their expertise, it was through the pandemic”* (Participant A).

These disparities fueled feelings of frustration and resentment among staff, underscoring the need for equitable recognition and support.

The return to work presented further challenges, with participants voicing worries about burnout and advocating for structured debriefing sessions to cope with the emotional strain of the pandemic,

*“Transitioning back to work was a little bit hard…It felt like all of a sudden we have to be here and we have to be performing again…Yes, it’s our work, but I felt like we kind of jumped in too fast and that caused burnout for some of us… We still had to take care of our own mental health too and get used to being back here… People didn’t really realize, I think, how impactful it was on everyone’s mental health.”* (Participant L)

Return-to-work pressures were similar in some ways to the general population. However, not all effects were shared equally by the community. Lower rates of remote options among Indigenous workers, due to differences in job mix, slowed recovery for women and young adults, and overcrowded housing and lack of broadband presented additional barriers to hybrid work and schooling from home for some homes in the community. These factors added stress and affected the timing of a steady return for some households. Human resources emerged as a critical focal point in navigating these challenges, necessitating a re-evaluation of traditional workplace dynamics and support systems.

Jurisdictional complexities surfaced as well, with participants pointing out conflicting advice from authorities and queries about the reach of each government authority within the First Nations community,

*“We did some tabletop exercises, but this was a different animal. We weren’t prepared, I don’t think for that [the pandemic] … We had a pandemic plan… Everything was just coming down from the guidelines from the Ontario government that we just kind of followed that.”* (Participant J)

While community members generally followed the guidelines, participants noted some pushback during the lockdown due to jurisdictional ambiguity and inability to enforce the measures. Nonetheless, the collective resilience and adaptability displayed by participants highlighted the community’s ability to withstand the pandemic’s impact and grow stronger. Ongoing reflection and adaptation will be pivotal in navigating future challenges and fostering a more resilient organizational landscape.

### Lessons learned and future planning

This category adopts a forward-looking approach, exploring the insights gained from the pandemic and exploring how community members and organizations are strategizing for future pandemics or similar scenarios, drawing from their experiences.

The concept of adaptation was frequently discussed by participants as they spoke of how their initial plans were revised as the situation evolved to best protect the community. For instance, one participant noted, “*Because it [the pandemic] was so dynamic, and it [the response] was all moving right is that they were able to adapt to what they needed to adapt to … let us do what we need to do to stay safe”* (Participant D). Participants spoke to the strong leadership and support received by the Health Director, Chief, and Band Council to enable staff to access and distribute required supplies, upskill, and fill in other roles as needed, and stay current with the latest information.

Future pandemic planning should prioritize health promotion, with community members at the forefront of these initiatives. Recognizing the significance of community-based platforms like the community Facebook page and newsletter, they were acknowledged as essential tools for disseminating knowledge and providing education:

*“We have a [newsletter] that goes out… I think for everybody else; Facebook was the go to. And then I think the ones, the seniors that didn’t have it, they had, you know, family that would kind of keep them up to date”* (Participant G);

*“We kind of took a step back and just started doing things more kind of on our own and sharing less [federal and provincial guidelines] … Just simplifying it and you know, putting it under our header or like everything was kind of coming from us… Even if it was word for word verbatim what they were saying, it just, it made a big difference”* (Participant F);

*“I knew community members, some of them didn’t have phones or didn’t have access to the Internet, so they couldn’t like see the online information that we were putting out. Early on in the pandemic we implemented our community newsletter again, so that was able to we were able to get information out. So that worked really well.”* (Participant E)

Participants frequently spoke of tailored communication strategies through these channels to ensure that community-members were educated on the benefits of obtaining vaccines and taking proper safety precautions, as opposed to feeling forced,

*“For the future… I think it’s just trying to educate people the best way that you can. If there is vaccines, educate about the benefits of the vaccine, but not be forceful. I feel like the forcefulness was wrong., and it put a divide in between people, and it was like it made nobody better than the other person”* (Participant C);

*“The First Nation didn’t make any of that [vaccines] mandatory, so I think the majority of people in the community saw that it was something that was going to help them… I feel like we had a good response to the vaccine and the convenience of having it here in our community and the ability for us to help our community members who may have been wondering, or anxious or unsure, they had people that were familiar. That they could speak to and find out more information… I think locally it was good thing that we were able to provide that here.”* (Participant I)

Additionally, the collaboration between community staff and public health unit personnel proved effective in fostering trust and comfort among vaccine recipients. Establishing trust and providing innovative supports for the vaccine programs contributed to their success, evident in an almost 80% vaccination rate for first and second doses among adults. As one participant noted,

*“Most of our families were happy to get vaccinated, and I found that they did a really good job down here with that, especially with the kids… They definitely had like snacks and drinks. They got to pick from a treasure chest. They had, like a little thing that vibrated to put on their skin [Buzzy]. They had the…therapy dogs were available, and it was it was fun for them… Our nurses, it was all local nurses that were giving the vaccinations, which is also nice because it’s a friendly face.”* (Participant D)

Giving communities autonomy to lead their pandemic response facilitated the implementation of diverse solutions, enhancing the effectiveness of the vaccine program. This autonomy was also cited as a key factor contributing to the successful management of COVID-19 within the community,

*“That [vaccine program] was really good because of the way that [Lambton] public health worked with us… They pretty well gave us the option of like we’ll do everything for you, if you want. However, much control you want, you can have it. So, they handled the hard part of getting the vaccine… We came up with the plan of like the layout, all the staff that were going to be there, all the bookings, and we just followed the guidelines to come up with the priority populations… Even though we were a priority population, we still had to kind of prioritize within that very first set of vaccines that came out… That was one thing that we took to council, and everybody was fine with the rules that we came up with.”* (Participant H)

Empowering communities to lead their pandemic response not only enhanced the success of vaccination programs but also played a crucial role in successful COVID-19 management. Participants stressed adapting to technology limitations, emphasizing resource provision like tablets for at-home appointments. This underscores the need for future funding to support virtual platform transitions. Additionally, a suggested hybrid return-to-work model includes rotating schedules for social distancing and access to office amenities, reflecting learned efficiencies from the pandemic.

The importance of a well-developed and effectively implemented pandemic plan was underscored; however, some participants expressed feeling not adequately prepared for the mitigation measures as what happened in reality was far from what they had envisioned when drafting the pandemic plan. Others emphasized the advantages of having a comprehensive emergency management plan already established and in place, which enhanced the community’s sense of readiness:

*“We have like an emergency notification system that you can sign up for and it goes out like via text or phone call kind of things. So, I know we utilized that a couple times for, you know, some urgent things [for the pandemic] … so that people know when they’re getting a phone call or something, it’s important”* (Participant F);

*“Soon as we declared an emergency, our Emergency Management plan should have kicked in, which there’s a piece in there on food… The Emergency Management plan is built for like evacuations or like disasters… So providing food for people switches over to another department in the Emergency Management plan where we have the Food Bank.”* (Participant H)

One facet of pandemic planning identified for enhancement revolved around enforcement and the allocation of responsibilities for implementing established measures. Participants also underscored challenges stemming from the tight-knit nature of the community, which impeded stringent enforcement of measures. Many situations discussed by participants required intervention to enforce lockdown or isolation measures.

To mitigate some of these issues involving multiple stakeholders, including broader agencies and public health units, in pandemic planning is crucial for future preparedness. For example, within the community they offer kindergarten to grade 2 locally, while most students continue their education in the public school system through Sarnia and the Lambton Kent District School Board. Participants stated that collaborating with the school board enabled them to align health protocols, ensuring consistency in screening tools and procedures for students. Involving multiple stakeholders was discussed as an important aspect of the pandemic response, “*I think our working group was very important and it’s a good practice because, having those different voices around the table, public works, education, health, administration, HR like we were able to from our own areas of expertise, able to share and support each other.”* (Participant I). Overall, the community’s pandemic response underscored the significance of collaboration and engagement with multiple stakeholders. By including broader agencies and public health units in planning efforts, communities to enhance their readiness for future challenges.

## Discussion

This study examines the unique experiences and obstacles encountered by a specific urban First Nations community in southwestern Ontario, Canada, spanning issues such as well-being and mental health, organizational dynamics, community and social factors, and considerations for future pandemics and adaptability. Expanding on these observations, we aim to explore these insights and extract practical lessons from the community’s experiences, enhancing the preparedness for future pandemics.

The COVID-19 pandemic had broad short-term impacts on communities, with the longer-term effects remaining uncertain. It prompted the implementation of unprecedented mitigation measures, such as nationwide lockdowns and vaccine mandates in Canada, which evolved rapidly as the situation changed. While effective, these measures, coupled with the broader impacts of the COVID-19 pandemic, exacerbated pre-existing vulnerabilities within the community and gave rise to new ones. Within our discussions, participants highlighted increased psychological stress, including “initial fear”, associated with the pandemic, aligning with global research indicating heightened rates of anxiety and depression [37–39]. Participants voiced concerns about the pandemic’s impact on the mental health and well-being of their community members, especially among youth, Elders, and individuals already grappling with mental health challenges, aligning with existing literature on the broader impacts of the pandemic [2,40–42]. One contributing factor to this phenomenon was the resemblance between pandemic measures and certain aspects of the historical and intergenerational trauma experienced by Indigenous peoples [43,44]. For example, lockdowns limited the social interactions amongst Indigenous peoples, echoing historical restrictions on leaving reserves [42]. Similarly, guidance by broader governments discouraging participation in ceremonies led to a perception of government control over Indigenous cultural practices, also echoing historic policies aimed at forced assimilation [42]. Building upon the insights provided by studies such as Gonzalez and Stewart [43], which explains how historical trauma contributes to ongoing health disparities among Indigenous populations, potentially exacerbating negative influences on healthcare decision-making during the pandemic, participants often discussed how this impacted the decision-making of community members. They noted that some community members disregarded lockdown measures, spreading misinformation, and having a reluctance to get vaccinated against COVID-19 may have been to historical trauma although other complex social factors also applied. Therefore, it is imperative to integrate efforts aimed at acknowledging and addressing historic and intergenerational traumas, along with endeavors to mitigate the negative impacts associated.

As a proposed solution, participants often discussed the advantages of having community-based staff lead initiatives such as the broader pandemic response, vaccination programs, and the dissemination of education and information directly from the community through platforms like the local newsletter or community Facebook page. This approach aims to foster trust and promote positive, education-focused messaging, contrasting with what some community members perceive as coercive communications from provincial and federal government sources. The significance of trusting relationships and robust communication to support awareness was underscored across several participants’ experiences. These findings are in line with community-based models of public health, which stress the importance of effective communication and local partnerships [45–47].

Another benefit of a community-led response was the increased awareness of prevalent social issues exacerbated during COVID-19 that needed attention within the community. Balancing work and family life also presented challenges, particularly during the lockdown periods with adults working and children attending school remotely. These sentiments parallel wider research on the challenges of telecommuting and online education [48,49]. Another concern which participants frequently discussed was the issue of food security. Various innovative programs, such as gardening initiatives, produce boxes, and virtual cooking programs, were discussed as effective means to address these challenges. Consistent with prior research, the pandemic worsened food insecurity, particularly affecting Indigenous peoples who already faced disproportionate rates [2,41,42]. As evidenced by our study and others, there is a need for increased funding to support community-driven solutions that mitigate food security impacts for future pandemics and other large-scale emergencies [50,51].

Other social factors highlighted by participants were substance-related harm and experiences of domestic violence, echoing previous literature [41,52,53]. It’s notable that the urban location of this First Nations community facilitated resource sharing, access to supplies and services, and coordinated response measures through the local public health unit and hospital. This contrasts with the added challenges faced by many remote and isolated First Nations communities during pandemics due to limited transportation and difficulties accessing supplies and resources [17]. Moreover, issues surrounding socioeconomic factors, such as food security, housing, and financial stability, were reported by participants. These findings are congruent with existing literature that suggests that socioeconomic factors played a significant role in pandemic experiences [54,55]. Furthermore, the lack of availability of affordable housing and issues of overcrowding are already prevalent issues within First Nations communities. Stay-at-home measures exacerbated the impact on mental health and contributed to risk factors linked to domestic violence [42,56]. Although the stay-at-home measures were crucial in preventing the spread of COVID-19, it is important to acknowledge and provide additional support for the further complexities created by those measures.

The success of any public health campaign largely depends on how effectively it communicates its objectives and methods, as well as how well these measures align with the community’s cultural beliefs and practices [57]. Therefore, the findings underscore the necessity for a robust, transparent, and multi-channel communication strategy for crisis management. Social media, particularly Facebook, emerged as the primary method of communication and proved highly effective in disseminating messages and countering misinformation when led by a trusted community organization. Participants frequently reflected on strategies that contributed to increased vaccine uptake, such as providing support for youth (e.g., therapy dogs, pain management tools), and having community members lead the vaccine program, including sharing photos and information about the clinics through community-specific communication channels. Similarly, in Maningrida, a community in the Northern Territory of Australia, vaccine uptake was increased by harnessing Indigenous leadership and self-governance to develop and implement tailored community-based and culturally appropriate strategies. These included sharing messaging in local languages via social media, organizing public town hall meetings, conducting door-to-door outreach for community members, and having Elders share messages of support within the community [44]. These examples underscore the significance of valuing, respecting, and upholding Indigenous social-governance systems to effectively tackle health inequities among Indigenous communities during future pandemics.

Jurisdictional challenges were raised by participants that presented barriers to an effective pandemic response. Similar to First Nations communities in the northwest region of Saskatchewan, Canada [58], our research illustrates ways in which strong Indigenous leadership and a pre-existing partnership with the local public health unit, which was strengthened during the response, helped to overcome jurisdiction complexities to deliver appropriate care to community members. In Canada, under the Constitution Act (1867), the provision of healthcare to the public is the responsibility of the provincial and territorial governments while the federal government is responsible for reserves [59,60]. The Indian Act (1982) asserts oversight of the federal government on reserves; however, newer legislation has enabled First Nations to control some or all the health services delivered in their communities [60,61]. Therefore, in Canada, federal, provincial/territorial, and First Nations governments can share responsibility for health services. This tripartite structure has proved to be problematic with regard to providing healthcare for First Nations, particularly during a pandemic, due to a lack of coordination and unclear roles and responsibilities between governmental bodies [17]. Thus, there is an imperative need for structures that overcome these “colonial fault lines” before the next pandemic [61]. Our study supports the strength of Indigenous-led public health efforts during the COVID-19 pandemic and calls for Indigenous sovereignty, autonomy, and self-determination during public health emergencies so that preparedness and response measures are grounded in their worldviews and realities [14,18,21,44,54,61].

### Future pandemic planning

It is important that we begin preparing for the future pandemics and incorporate these learnings from previous pandemics to improve health and wellbeing outcomes for First Nations communities. The wide-ranging impacts of a pandemic underscore the need for pandemic plans to be more holistic and comprehensive. Participants emphasized the need for better preparedness and adaptability. These sentiments support the existing literature advocating for community resilience and emergency preparedness [62,63].

Many pandemic plans continue to entail mitigation, preparedness, response, and recovery activities for the health sector. However, our research, in addition to others, notes that pandemics cause widespread impacts beyond the health sector particularly in relation to employment, education, food security, substance-related harm, domestic violence, and housing. Indigenous determinants of health have been conceptualised as distal (e.g., historical, and ongoing impacts of colonialism, racism, social exclusion), intermediate (e.g., inadequate education, healthcare services, and community infrastructure), and proximal (e.g., food insecurity, employment, and income) [26]. Our study provides empirical support for theories emphasizing the importance of social determinants of health, such as socioeconomic status and educational attainment, in influencing the experience of crises [64]. Pandemic preparedness must sufficiently encompass the social and cultural determinants that impact Indigenous health. This means that all government levels must address issues related to secure housing and employment, food security and emergency food programmes, as well as access to appropriate healthcare and technology before the next pandemic. Educational and corporate institutions need to develop more flexible and adaptive systems to cater to remote working and learning challenges. Importantly, given the substantial psychological distress and increased cases of substance use and domestic violence reported, there must be increased support for tailored Indigenous-led mental health and wellbeing initiatives and programmes to reduce substance-related harm as we recover from the COVID-19 pandemic and prepare for future pandemics [6,65].

**Fig 1:**
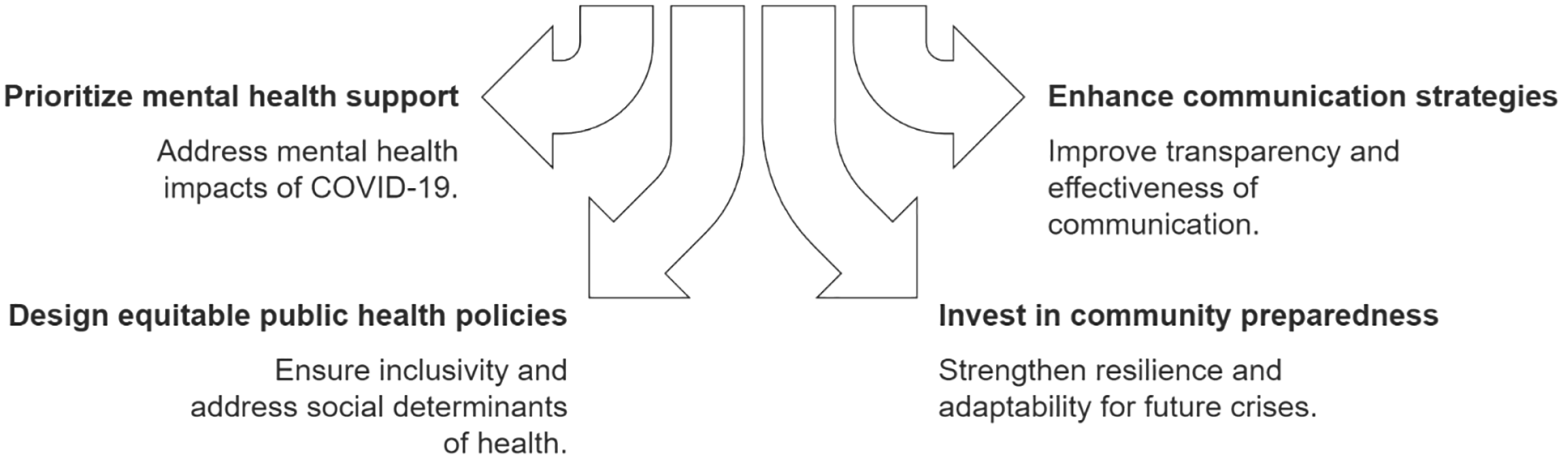
Key areas for enhancing pandemic response in First Nations communities.

It is imperative to reduce health risks associated with emergencies and disasters by focusing on prevention and preparedness (rather than on reaction) and improving coordination within and across sectors [29]. The WHO recently developed the Health Emergency and Disaster Risk Management (Health EDRM Framework), which calls for a shift towards an approach that is risk-based, proactive, addresses all-hazards, vulnerability and capacity focused, includes whole-of-society, shares responsibility, manages risk, and plans with communities [29]. Rather than developing separate, stand-alone response plans for each hazard, the Framework calls for plans to address common issues with similar health risks and include additional risk-specific information as needed [29]. All previous pandemics have been caused by the influenza virus except for the recent COVID-19 pandemic which was caused by a coronavirus [66]. Being respiratory pathogens, they all share some similar transmission dynamics meaning that aspects of preparedness and response efforts can be generalised. However, as the COVID-19 pandemic response saw the implementation of unprecedented mitigation measures (e.g., nation-wide lockdowns, vaccine mandates), it is important to include specific guidance based on risk. As such, the WHO’s new Preparedness and Resilience for Emerging Threats Initiative (PRET) also calls for improvements to disease pandemic preparedness by leveraging this shared understanding to develop integrated plans [67]. While the ‘all-hazards’ paradigm can be considered appropriate to guide the COVID-19 pandemic response (Penta et al., 2021), others have argued the flaws in unified planning given that pandemics are unique in nature [68]. Given the importance of effectively and efficiently using resources within First Nations communities and the multiple hazards they face, pandemic planning could be re-conceptualised to evolve from single-event plans to a comprehensive health emergency and disaster risk management plan that integrates a wider range of events with similar health risks while prioritizing the likelihood and severity based on their specific local circumstances. Fig 1 highlights key areas where pandemic response efforts in First Nations communities can be enhanced. Ultimately, with these enhancements, we envision self-governance at the centre with principles of coordinated partnerships (i.e., Lambton Public Health, school board, hospital), transparent and noncoercive communication, operational supports (food security, wellness checks, hybrid care), workforce and business continuity, and jurisdiction navigation, with each core element and operational action aligned to the principles and practices of Health-EDRM/PRET for all-hazards preparedness.

Existing research has detailed disproportional impacts and historic drivers of inequity; less widely shared are urban, implementation-detailed case studies, led by Indigenous governance, which unpack how trust, communication, and cross-jurisdictional coordination were made real. Herein, we document replicable practices (e.g., community-run communications, culturally safe vaccination workflows, and rapid pivots to meet social-support needs) and link these to all-hazards emergency management guidance to inform next steps beyond COVID-19 [44].

### Limitations

While this research utilized a community-based participatory approach, gathering insights from key informants such as the pandemic committee and frontline workers, the findings may not be broadly generalizable beyond the specific urban First Nations community in Ontario, Canada. Although the qualitative approach provided rich contextual information on the social processes of managing a public health emergency, it is essential to recognize the inherent diversity among Indigenous Peoples and First Nations communities. Furthermore, given the scarcity of research on pandemic planning tailored to these populations, this study contributes to the literature by highlighting the crucial role of community-led approaches in addressing the unique needs of structurally disadvantaged groups. However, it is important to note that the findings captured experiences at a single point in time just after the WHO declared the pandemic over. Longitudinal studies would offer a more comprehensive understanding of the longer-term effects of the pandemic and necessary adaptive strategies. Finally, in keeping with being an urban First Nation with a close relationship with municipal services, some operational facilitators may be different from remote and northern contexts; our role of this research is to help bridge this urban evidence gap [69].

## Conclusion

The findings of this study contribute to broadening our understanding of the diverse impacts of the COVID-19 pandemic on First Nations communities, offering unique insights gathered from an urban First Nations community. Our results underscore the need for multi-dimensional approaches to managing public health emergencies, incorporating mental health and socio-economic support, and effective partnership and communication strategies. Building upon a qualitative exploration of community experiences, this research examined five primary thematic realms: well-being and mental health, work-life balance, community and social factors, organizational dynamics, and lessons learned. Within these themes, significant challenges emerged, including impacts on mental health and well-being, food security, socioeconomic obstacles, and disruptions in work-life balance and education. The importance of robust and transparent communication channels and the need for better preparedness and adaptability were also highlighted. The study’s findings offer significant contributions to understanding community responses to pandemics and crises. They underscore the necessity for multi-pronged approaches in pandemic management, emphasizing the importance of mental health support, effective communication, and inclusive public health policies. Recommendations stemming from the findings include the urgent need for targeted mental health services, the development of robust communication strategies, the design of equitable public health policies, the creation of flexible education and work-life balance systems, and the investment in comprehensive community preparedness plans. By targeted mental health services, we are referring to community-led supports aligned to priority populations (youth, Elders, people with substance-use issues, bereaved families) offered in person when required or for more complex needs and maintained virtually where possible, virtual counselling, visits with traditional healers, school wellness checks, and Jordan’s Principle–facilitated referrals. Importantly, the findings call for future pandemic planning efforts to support Indigenous leadership, address the social and cultural determinants of Indigenous health, and move towards a comprehensive approach.
